# WHO Global Rotavirus Surveillance Network: A Strategic Review of the First 5 Years, 2008–2012

**Published:** 2014-07-25

**Authors:** Mary M. Agócs, Fatima Serhan, Catherine Yen, Jason M. Mwenda, Lúcia H. de Oliveira, Nadia Teleb, Annemarie Wasley, Pushpa R. Wijesinghe, Kimberley Fox, Jacqueline E. Tate, Jon R. Gentsch, Umesh D. Parashar, Gagandeep Kang

**Affiliations:** 1 Department of Immunization, Vaccines, and Biologicals, World Health Organization (WHO), Geneva, Switzerland; 2 Division of Viral Diseases, National Center for Immunization and Respiratory Diseases, CDC; 3 Global Immunization Division, Center for Global Health, CDC; 4 WHO Regional Office for Africa, Brazzaville, Republic of the Congo; 5 WHO Regional Office for the Americas, District of Columbia, United States; 6 WHO Regional Office for the Eastern Mediterranean, Cairo, Egypt; 7 WHO Regional Office for Europe, Copenhagen, Denmark; 8 WHO Regional Office for South-East Asia, New Delhi, India; 9 WHO Regional Office for the Western Pacific, Manila, Philippines; 10 Christian Medical College, Vellore, India

Since 2008, the World Health Organization (WHO) has coordinated the Global Rotavirus Surveillance Network, a network of sentinel surveillance hospitals and laboratories that report to ministries of health (MoHs) and WHO clinical features and rotavirus testing data for children aged <5 years hospitalized with acute gastroenteritis. In 2013, WHO conducted a strategic review to assess surveillance network performance, provide recommendations for strengthening the network, and assess the network’s utility as a platform for other vaccine-preventable disease surveillance. The strategic review team determined that during 2011 and 2012, a total of 79 sites in 37 countries met reporting and testing inclusion criteria for data analysis. Of the 37 countries with sites meeting inclusion criteria, 13 (35%) had introduced rotavirus vaccine nationwide. All 79 sites included in the analysis were meeting 2008 network objectives of documenting presence of disease and describing disease epidemiology, and all countries were using the rotavirus surveillance data for vaccine introduction decisions, disease burden estimates, and advocacy; countries were in the process of assessing the use of this surveillance platform for other vaccine-preventable diseases. However, the review also indicated that the network would benefit from enhanced management, standardized data formats, linkage of clinical data with laboratory data, and additional resources to support network functions. In November 2013, WHO’s Strategic Advisory Group of Experts on Immunization (SAGE) endorsed the findings and recommendations made by the review team and noted potential opportunities for using the network as a platform for other vaccine-preventable disease surveillance. WHO will work to implement the recommendations to improve the network’s functions and to provide higher quality surveillance data for use in decisions related to vaccine introduction and vaccination program sustainability.

## Background

Rotavirus is a leading cause of severe gastroenteritis among children aged <5 years worldwide, accounting for approximately 5% of child deaths annually (1). Since 2009, WHO has recommended that rotavirus vaccines be included in all national immunization programs, particularly in countries with high diarrhea-related child mortality (2). As of April 2014, a total of 56 (29%) of 194 WHO member states had introduced rotavirus vaccine, with 20 (36%) of those countries eligible for financial support from the GAVI Alliance, a public-private global health partnership that has helped increase access to immunization in poor countries (3–4). Key factors for countries to consider in the decision to introduce rotavirus vaccine include local burden, trends, and age distribution of disease (5). Disease surveillance systems can play a key role in providing such information and potentially serve as platforms for impact assessments after vaccine introduction.

In 2008, WHO brought together existing regional surveillance networks to establish a standardized global sentinel hospital surveillance network for rotavirus disease, with financial support from the GAVI Alliance. The Global Rotavirus Surveillance Network includes sentinel surveillance hospitals and laboratories that report to MoHs and WHO clinical features and rotavirus testing data for children aged <5 years hospitalized with acute gastroenteritis. In addition to managerial oversight, WHO provides technical assistance to countries, as well as financial support to countries eligible for GAVI Alliance funding for surveillance activities. During the prevaccination introduction period, original objectives of the network were to 1) provide data for describing disease epidemiology, including disease burden, 2) establish a platform to measure impact after vaccine introduction, and 3) identify circulating strains and strain distribution. Objectives after vaccine introduction were to 1) assess disease trends, 2) monitor changes in circulating strains, and 3) use the platform for vaccine effectiveness studies.

During 2008–2012, WHO established a network of sentinel hospital and national laboratories supported by regional reference laboratories and a global reference laboratory. Additionally, WHO launched an annual external quality assessment program targeting participating laboratories, developed a standardized protocol for sentinel site assessments, provided technical advice and laboratory supplies to sites, and shared data semiannually via a global surveillance and information bulletin.* In 2011, WHO established an informal Technical Advisory Group of experts for new vaccines surveillance (iTAG) and a laboratory technical working group to provide guidance for further improving and standardizing the global surveillance network. By 2012, the global network had expanded to 178 sentinel surveillance sites in 60 countries (72% of which were eligible for GAVI Alliance support) from all six WHO regions.

In 2013, WHO conducted a strategic review of surveillance network performance in the context of the recommendation for quality case-based disease surveillance in the 2011–2020 Global Vaccine Action Plan (6). The objectives of the review were to 1) assess whether and to what extent the 2008 objectives for the network were met, 2) assess MoH perspectives on the need and value of the network, 3) assess laboratory network management, 4) review existing data management systems, 5) assess the adequacy of resources available to WHO, and 6) provide recommendations for strengthening the network and assess the network’s utility as a platform for other vaccine-preventable disease surveillance.

## Structure of the strategic review

WHO, under the direction of iTAG and with guidance from technical partners, performed the following assessments to review surveillance network performance during 2008–2012: 1) questionnaires to obtain country-level expert and MoH staff opinions about the value of the surveillance data for supporting national decisions on vaccine introduction, 2) reviews of the laboratory network and data management systems by external consultants, 3) review of published literature and GAVI Alliance vaccine introduction applications to evaluate use of rotavirus surveillance data, and 4) internal review of WHO activities and funding disbursement. WHO also analyzed data from sentinel sites that reported ≥10 months of data and tested ≥100 stool specimens each year in 2011 and 2012. Data analysis for sites that met these inclusion criteria included description of the burden, trends, and age distribution of rotavirus disease, rotavirus genotype distribution, and rotavirus disease trends before and after vaccine introduction in countries that had sufficient data.

WHO and iTAG members discussed review methodology and preliminary findings during monthly teleconferences, leading to a comprehensive review of all findings during a September 2013 meeting. Findings and proposed actions to strengthen the surveillance network and further improve surveillance data quality and use were presented to SAGE in November 2013.†

## Summary of strategic review findings

Countries in all six WHO regions valued and used the rotavirus surveillance platform and data for vaccine introduction decisions, as well as for disease burden estimates and advocacy. In some cases, countries used the platform to conduct special studies to assess vaccination impact on rotavirus disease. During 2011–2012, a total of 169 sites in 55 countries reported rotavirus surveillance data for both years to WHO (Figure). Seventy-nine (47%) sites in 37 countries met the reporting and testing inclusion criteria for data analysis, including 63 (80%) sites in 32 countries eligible for GAVI Alliance support. Thirteen (35%) of the 37 countries with sites meeting inclusion criteria had introduced rotavirus vaccine nationwide; one had introduced rotavirus vaccine subnationally in a single province. Among sites included in the analysis, the median monthly percentage of eligible children enrolled in surveillance was 93%. The mean percentage of rotavirus detection among 75,353 tested children during January 2011–December 2012 was 36%, with the largest percentage positive (42%) in infants aged 6–11 months. The most frequently observed rotavirus genotypes during 2009–2012 were the five considered globally prevalent (G1P[8], G2P[4], G3P[8], G4P[8], and G9P[8]), although regional differences were observed.

The strategic review team concluded that sites included in the analysis were meeting the 2008 objectives for documenting presence of disease, describing disease epidemiology, using surveillance as a platform for special studies in some countries, and using the data for policy decisions. The majority of countries eligible for GAVI Alliance support and sites receiving financial support consistently met performance indicator targets for recruitment, testing, and reporting. The review team noted that the network would benefit from enhanced management to ensure accountability at all levels and noted that a lack of standardized data formats and incomplete linkage of clinical and laboratory data in some locations limited the network’s ability to undertake real-time performance monitoring and analyses. To fully support the global network, additional human and financial resources are needed at all levels to support management, data processing and analysis, and recommended on-site assessments, and also to support countries not eligible for GAVI Alliance support. Short-term, year-to-year funding inhibited longer-term program planning and investment at all network levels.

## SAGE-endorsed findings and recommendations

In November 2013, SAGE endorsed the strategic review findings and agreed that the experience of the network’s first 5 years should inform future surveillance needs, including potential use of the network as a platform for other vaccine-preventable disease surveillance (7). SAGE also noted that surveillance data will be essential to secure long-term national funding for rotavirus vaccines in some countries, and that demonstrating vaccine impact in epidemiologic settings not included in existing impact data is important (7). SAGE recommended the following:

1. revision of the surveillance objectives to align more closely with the current and future vaccine introduction landscape;
2. further standardization to ensure the generation of credible, well-defined data with linking of clinical and laboratory data at all levels and real-time monitoring of system performance;
3. sharing of standardized, case-based data at all levels; use of identifiers for linking of clinical and laboratory results; zero/negative reporting to differentiate absence of cases from lack of reporting; and progress on data management, including the use of software with editing and verification capability;
4. development of performance measures and agreements on a) sentinel site eligibility for ongoing participation in the network, b) standards for the reference laboratories, including site visits, conduct of specialized testing, and testing of a systematic sample of specimens from all sites for laboratory quality control, and c) WHO roles in support of the network, and
5. additional human and financial resources to strengthen the networks, through increased access to technical assistance, laboratory quality assurance/control processes, data management systems, exchange of lessons learned, and collaboration.

### Discussion

The WHO-coordinated Global Rotavirus Surveillance Network has met its original objectives of documenting and describing rotavirus disease burden and providing useful data for policy decisions. However, the network can be enhanced further and will require continual performance monitoring for it to be responsive to the changing information needs of all immunization stakeholders. WHO, under guidance of the iTAG and technical immunization partners, will work to implement the strategic review recommendations and to monitor sentinel hospital site and laboratory performance quarterly. WHO has developed a management framework that defines implementation activities and timelines. Implementation of the strategic review recommendations will improve the network’s functions and ability to integrate with other surveillance platforms, and will provide higher quality surveillance data for use in decisions related to vaccine introduction and vaccination program sustainability.

What is already known on this topic?

Rotavirus disease is a leading cause of severe diarrhea morbidity and mortality among children aged <5 years worldwide. The World Health Organization (WHO) recommends the inclusion of rotavirus vaccine in all national immunization programs. Disease surveillance systems provide data on local disease burden and epidemiology, which can play a key role in vaccine introduction decisions.

What is added by this report?

By 2012, the Global Rotavirus Surveillance Network included 178 sentinel surveillance sites in 60 countries. A network performance review determined that during 2011 and 2012, a total of 79 sites in 37 countries met reporting and testing inclusion criteria for data analysis. Of the 37 countries with sites meeting inclusion criteria, 13 (35%) had introduced rotavirus vaccine nationwide. All 79 sites included in the analysis were meeting 2008 network objectives of documenting presence of disease and describing disease epidemiology, and many countries were using the rotavirus surveillance data for vaccine introduction decisions, disease burden estimates, and advocacy. WHO’s Strategic Advisory Group of Experts on Immunization (SAGE) has endorsed recommendations to enhance network management, standardize data formats, link clinical and laboratory data, and provide additional resources to support network functions.

What are the implications for public health practice?

Implementing recommendations for strengthening the Global Rotavirus Surveillance Network will improve the network’s functions and ability to integrate with other surveillance platforms, and will provide higher quality surveillance data for use in decisions related to rotavirus vaccine introduction and vaccination program sustainability.

## Figures and Tables

**FIGURE:**
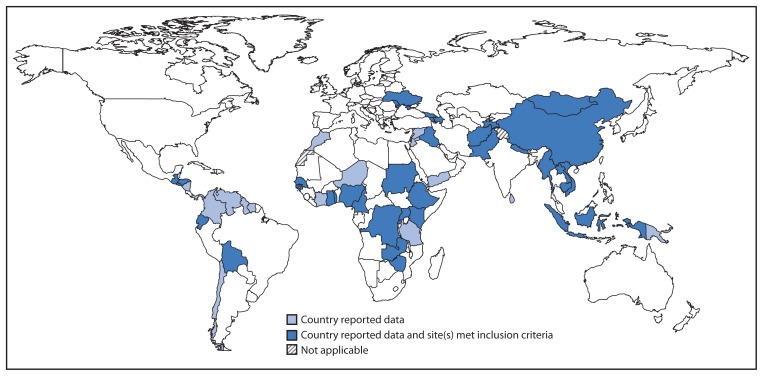
Reporting countries and countries with sentinel sites meeting inclusion criteria — World Health Organization, Global Rotavirus Surveillance Network, 2011–2012

## Footnotes

* Available at http://www.who.int/immunization/monitoring_surveillance/resources/NUVI.

† Additional information about SAGE is available at http://www.who.int/immunization/sage.
